# Ballantyne Syndrome Related to Down Syndrome and Chorioamnionitis: A Case Report and Literature Review

**DOI:** 10.7759/cureus.78563

**Published:** 2025-02-05

**Authors:** Alonso J Garcia, Pedro Vega, Gabriel R Chacón, Christian Silva Rengifo

**Affiliations:** 1 Medicine, Universidad Peruana de Ciencias Aplicadas, Lima, PER; 2 Obstetrics and Gynecology, Hospital Nacional Guillermo Almenara Irigoyen, Lima, PER

**Keywords:** ballantyne syndrome, chorioamnionitis, edema, fetal hydrops, trisomy 21

## Abstract

Ballantyne syndrome is a rare obstetric condition characterized by fetal hydrops, maternal edema, and placentomegaly. Its low incidence, combined with potential clinical overlap with preeclampsia, poses significant diagnostic and management challenges. This condition is especially relevant due to the severe complications it can cause during pregnancy.

In this article, we present a case of Ballantyne syndrome associated with Down syndrome (DS) and chorioamnionitis, complemented by a literature review exploring its possible etiologies, pathophysiology, and therapeutic options. Additionally, the importance of early diagnosis, the use of invasive techniques to identify underlying etiologies, and therapeutic decision-making focused on safeguarding maternal life are emphasized, including pregnancy termination in cases of severe complications to optimize maternal and fetal outcomes.

## Introduction

Ballantyne syndrome is a rare obstetric pathology that is characterized by the triad that includes fetal hydrops, maternal edema, and placentomegaly [1]. This syndrome is also known as "mirror syndrome," since the changes that occur in the fetus are reflected in the mother [2]. Since 1892, the year in which John W. Ballantyne described this syndrome for the first time, numerous attempts have been made to establish the mechanisms of its pathogenesis. To date, this syndrome has been linked to a molecular process involving endothelial dysfunction such as increased levels of soluble fms-like tyrosine kinase 1 (sFlt1) and immune components due to reports of cases associated with infection by cytomegalovirus, parvovirus B19, Coxsackie virus, and Rhesus (Rh) isoimmunization [3-5]. Additionally, a case related to Down syndrome (DS) has been reported where the newborn was initially alive but passed away a few hours later. Trisomy 21, the most common cause of hydrops, may also lead to Ballantyne syndrome if mirrored by the mother [6]. Down syndrome affects one in 319 to one in 1,000 babies, depending on maternal age and prenatal screening schedules [7]. This enables early detection, providing opportunities for informed decision-making and improved pregnancy management [8].

Among the clinical findings of this syndrome, edema, gestational hypertension, proteinuria, hypoalbuminemia, ascites, maternal weight gain, dyspnea, and hemodilution anemia stand out [9]. Several of these manifestations are similar to those presented in preeclampsia [10]. It has been proposed that Ballantyne syndrome shares molecular processes with preeclampsia, which include endothelial dysfunction, oxidative stress, and the imbalance between pro-angiogenic and antiangiogenic factors [3]. This could explain why the number of reported cases is underestimated due to the overlap between both conditions.

Due to the extremely rare nature of this syndrome, with less than 120 cases described in the literature to date, an exact description of its clinical presentation, diagnostic approach, and management remains insufficient [11].

This report presents the case of a 29-year-old patient at 31 weeks of gestation who sought medical attention due to uterine dynamics. During an emergency evaluation, polyhydramnios was identified, and Ballantyne syndrome was diagnosed during hospitalization.

## Case presentation

A 29-year-old primigravida patient with no significant medical or surgical history had a genetic ultrasound performed outside our institution during the first trimester, as well as ultrasounds at weeks 25 and 29 of gestation, all of which were reported without abnormalities. She presented to the emergency department at 31 weeks of gestation due to uterine dynamics. The physical examination revealed an increased fundal height (FH) for gestational age (FH: 38 cm). Due to the persistence of uterine dynamics, hospitalization for further evaluation was deemed necessary. Tocolysis was initiated with nifedipine in a loading dose (20 mg every 20 minutes for three doses), maintenance of 10 mg every six hours, and fetal maturation with dexamethasone.

**Figure 1:**
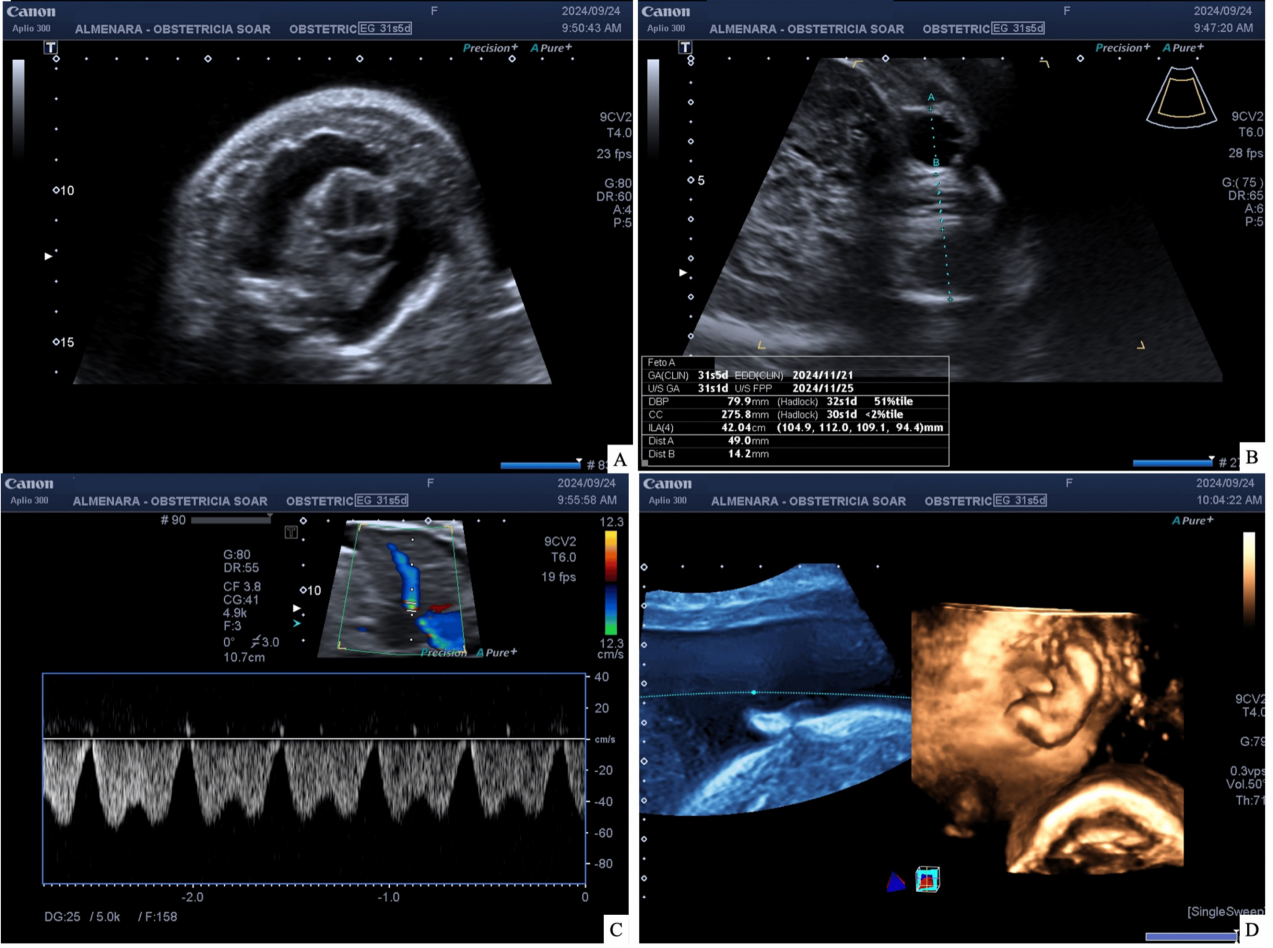
Fetal ultrasound (A) Four cavities and normal outflow tracts, bilateral hydrothorax, and no tricuspid regurgitation (study limited by polyhydramnios); (B) hypotelorism; (C) reverse flow in the ductus venosus; (D) edema of the skin and subcutaneous tissue

**Figure 2:**
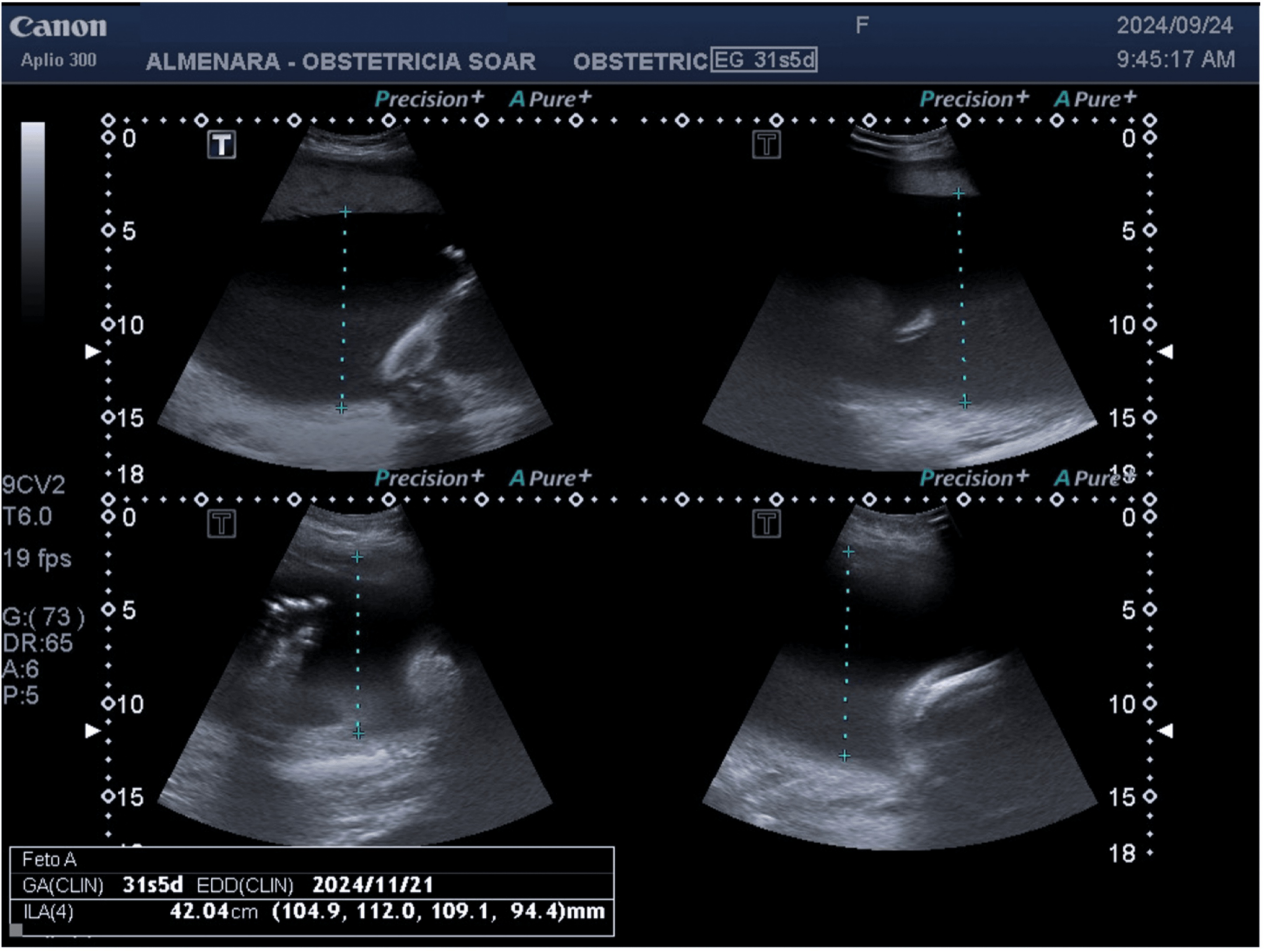
Severe polyhydramnios Ultrasound demonstrates a fetus with findings suggestive of severe polyhydramnios (amniotic fluid index: 42.04 cm)

An obstetric ultrasound was performed during which an active single fetus, severe polyhydramnios with an amniotic fluid index (AFI) of 41 cm, fetal hydrops, and reverse flow in the ductus venosus were reported. In addition, micromelia suggestive of lethal skeletal dysplasia was identified, along with a possible pathology of ventral induction due to hypotelorism, which posed a worse fetal prognosis (Figures 1, 2). Given this situation, a chromosomopathy was considered, and amniodrainage was performed both as a treatment for polyhydramnios and to facilitate a fetal karyotype study. The follow-up ultrasound after drainage revealed an AFI of 30 cm with a vertical pocket exceeding 9 cm, situs solitus, and pericardial effusion accompanied by findings suggestive of cardiac diastolic dysfunction. The toxoplasmosis, other agents, rubella, cytomegalovirus, and herpes simplex (TORCH) test was negative. 

After four days of admission to the emergency obstetrics department, the vital functions remained within normal parameters; however, leukocytosis (20,650/mcL), neutrophilia (87%, 17,97×10^3^/µL), hypoalbuminemia (2.64 g/dL), elevated transaminases, and generalized edema predominantly of the lower extremities were present; due to maternal deterioration and fetal prognosis, the termination of the pregnancy through an emergency cesarean section was performed. A live female newborn was obtained with an appearance, pulse, grimace, activity, and respiration (APGAR) score of 5 at one minute and 8 at five minutes and a weight of 2,245 g. The patient received antibiotic treatment with ceftriaxone 2 g every 24 hours after delivery. Subsequently, the edema decreased, and the blood count remained within adequate limits; she was discharged seven days after the cesarean section.

**Figure 3:**
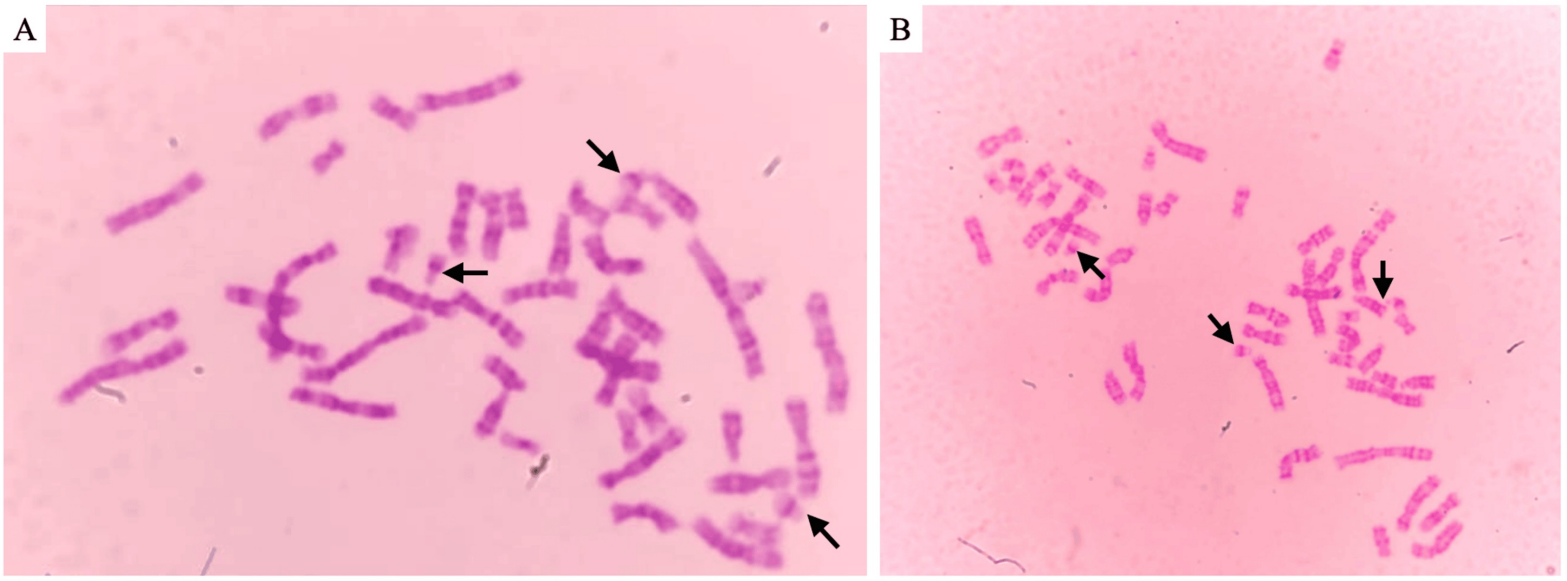
Cytogenetic study (A and B) Amniotic fluid chromosome slide showing the diagnosis of trisomy 21, a 47,XX,+21 karyotype confirming Down syndrome. The three chromosome 21 are indicated with arrows. The analysis was performed with a resolution of 400 bphs using GTG banding of cultured amniocytes bphs, bands per haploid set; GTG, G-bands by trypsin using Giemsa

During postpartum follow-up, the cytogenetic study of amniotic fluid revealed the most common cytogenetic type of DS: complete free trisomy, a 47,XX,+21 karyotype (Figure 3). After receiving the result, the family was counseled by a psychologist and psychiatrist to help them process the diagnosis of aneuploidy and provide emotional support. The gynecologist explained the implications of the diagnosis and discussed available options; no representative from the genetics department was available to provide additional insight. They were also informed about the potential risks and management for future pregnancies, including prenatal genetic screening options for subsequent pregnancies. To this, the family responded that this would be the only pregnancy with such considerations and that no further pregnancies were planned. The newborn passed away and was transferred to the department of pathology for further examination, although no additional information was obtainable. Additionally, the histological study of the placenta identified microabscesses and necrosis on the fetal surface, which suggested acute chorioamnionitis; it also presented poor maternal vascular perfusion with recent infarcts and accelerated villous maturation, indicative of an ascending intrauterine infection.

## Discussion

Ballantyne syndrome is an extremely rare entity, with less than 120 reported cases in the last 40 years [12]. It is estimated to complicate 50% of fetal hydrops cases and occurs in one in 6,000 pregnancies [13]. This syndrome usually manifests between weeks 16 and 34 of gestation and is characterized by the presence of placental-fetal edema [13,14]. In addition, it presents other symptoms such as high blood pressure, maternal hemodilution, albuminuria, proteinuria, and slight elevation of liver enzymes [14]. Although it has a very low incidence, this syndrome is associated with severe maternal complications, with acute lung edema being the most frequent. Furthermore, it is related to a rate of intrauterine fetal death of 57.89% [12].

Currently, the etiology and pathophysiology of this syndrome remain unclear; only a few possible causes have been proposed so far. In the systematic review by Allarakia et al., the causes associated with this syndrome are Rhesus isoimmunization, fetal cytomegalovirus, and parvovirus B19 infection [12]. Furthermore, other causes are described such as fetal arrhythmias (e.g., fetal supraventricular tachycardia), placental chorioangioma, structural cardiac malformations (Ebstein), and fetal tumors [15].

In this case, it is proposed that Ballantyne syndrome was caused by a non-immune etiology since nearly 90% of cases of fetal hydrops are attributed to this cause. Among these, trisomy 21, a chromosomal abnormality (which was obtained in genetic testing), represents one of the most frequent causes of fetal hydrops, reinforcing its relevance as an etiological factor in this context [16,17]. Additionally, chorioamnionitis, a placental phenotype that contributes to approximately 33% of non-immune hydrops cases, is included among the four major processes defined by the Amsterdam criteria for the evaluation of placental pathology: maternal vascular malperfusion (MVM), fetal vascular malperfusion (FVM), acute chorioamnionitis, and chronic villitis of unknown etiology (VUE) [18,19]. In the literature, a similar case was described of a 36-year-old patient with Ballantyne syndrome during her third pregnancy, which resulted in intrauterine fetal death and subsequent birth of a neonate with fetal hydrops and also the presence of trisomy 21 [6].

The diagnosis of this syndrome should focus on identifying the underlying cause of fetal hydrops; this includes a detailed history, Doppler ultrasound (which should include middle cerebral artery Doppler for the evaluation of fetal anemia), and, if necessary, additional studies, such as karyotyping and the detection of fetal infections (including TORCH), using invasive techniques such as cordocentesis or amniocentesis [15]. The diagnosis is based on the presence of signs in both the mother and the fetus. Although simultaneous presence is expected, there may also be temporal variability in the appearance of signs with a difference of up to two weeks between the mother and the fetus or vice versa [12].

On this occasion, we present the case of a primigravida with no significant history who developed Ballantyne syndrome related to Down syndrome and underlying chorioamnionitis. This case underscores the relevance of early diagnosis and a multidisciplinary approach in managing this condition, as well as highlighting the importance of assessing the timing of delivery in cases of severe complications. It also highlights the necessity of prenatal screening as a crucial step before diagnosis through genetic testing. Given the high prevalence of Down syndrome, modern prenatal screening approaches, such as non-invasive prenatal testing (NIPT), now allow for the early identification of this and other common aneuploidies, offering families the opportunity to make informed decisions [8]. In Peru, screening is routinely offered. However, only the genetic ultrasound is covered by the healthcare system, even though all options are stated in the guidelines. Any other examination must be done privately (e.g., the cost of the pregnancy-associated plasma protein A {PAPP-A} test in our country is equivalent to one-third of the monthly minimum wage). Obstetric management primarily relies on clinical evaluation, Doppler ultrasound, fetal morphological assessment, and fetal-chromosomal analysis, rather than universal screening programs such as first-trimester combined tests or NIPT [20]. The review of the literature highlights the rarity of these cases, the challenges in determining the etiology, and the complexity of the approach, which is still marked by uncertainties regarding the pathophysiology and treatment.

## Conclusions

The case presented highlights the uniqueness of the possible relationship between Ballantyne syndrome, Down syndrome, and chorioamnionitis, although this connection still raises questions due to the limited evidence available. The literature review and case-specific findings demonstrate the need for interdisciplinary approaches to optimize maternal and fetal outcomes, especially in settings where early screening, diagnosis, and therapeutic decision-making are critical. Furthermore, the analysis reinforces the importance of considering uncommon etiologies in contexts of fetal hydrops and maternal deterioration. It is also strongly recommended to utilize prenatal screening methods to identify potential aneuploidies, as early detection may help in managing more complex cases effectively. All these characteristics invite future research to investigate further into the pathophysiology, screening options, and treatments of this very rare clinical entity.
